# Challenges and Future Directions of Big Data and Artificial Intelligence in Education

**DOI:** 10.3389/fpsyg.2020.580820

**Published:** 2020-10-19

**Authors:** Hui Luan, Peter Geczy, Hollis Lai, Janice Gobert, Stephen J. H. Yang, Hiroaki Ogata, Jacky Baltes, Rodrigo Guerra, Ping Li, Chin-Chung Tsai

**Affiliations:** ^1^Institute for Research Excellence in Learning Sciences, National Taiwan Normal University, Taipei, Taiwan; ^2^National Institute of Advanced Industrial Science and Technology, Tsukuba, Japan; ^3^School of Dentistry, Faculty of Medicine & Dentistry, University of Alberta, Edmonton, AB, Canada; ^4^Graduate School of Education, Rutgers – The State University of New Jersey, New Brunswick, NJ, United States; ^5^Apprendis, LLC, Berlin, MA, United States; ^6^Department of Computer Science and Information Engineering, College of Electrical Engineering and Computer Science, National Central University, Taoyuan City, Taiwan; ^7^Graduate School of Informatics, Kyoto University, Kyoto, Japan; ^8^Department of Electrical Engineering, College of Technology and Engineering, National Taiwan Normal University, Taipei, Taiwan; ^9^Centro de Tecnologia, Universidade Federal de Santa Maria, Santa Maria, Brazil; ^10^Department of Chinese and Bilingual Studies, Faculty of Humanities, The Hong Kong Polytechnic University, Kowloon, Hong Kong; ^11^Program of Learning Sciences, National Taiwan Normal University, Taipei, Taiwan

**Keywords:** big data, artificial intelligence, education, learning, teaching

## Abstract

We discuss the new challenges and directions facing the use of big data and artificial intelligence (AI) in education research, policy-making, and industry. In recent years, applications of big data and AI in education have made significant headways. This highlights a novel trend in leading-edge educational research. The convenience and embeddedness of data collection within educational technologies, paired with computational techniques have made the analyses of big data a reality. We are moving beyond proof-of-concept demonstrations and applications of techniques, and are beginning to see substantial adoption in many areas of education. The key research trends in the domains of big data and AI are associated with assessment, individualized learning, and precision education. Model-driven data analytics approaches will grow quickly to guide the development, interpretation, and validation of the algorithms. However, conclusions from educational analytics should, of course, be applied with caution. At the education policy level, the government should be devoted to supporting lifelong learning, offering teacher education programs, and protecting personal data. With regard to the education industry, reciprocal and mutually beneficial relationships should be developed in order to enhance academia-industry collaboration. Furthermore, it is important to make sure that technologies are guided by relevant theoretical frameworks and are empirically tested. Lastly, in this paper we advocate an in-depth dialog between supporters of “cold” technology and “warm” humanity so that it can lead to greater understanding among teachers and students about how technology, and specifically, the big data explosion and AI revolution can bring new opportunities (and challenges) that can be best leveraged for pedagogical practices and learning.

## Introduction

The purpose of this position paper is to present current status, opportunities, and challenges of big data and AI in education. The work has originated from the opinions and panel discussion minutes of an international conference on big data and AI in education (The International Learning Sciences Forum, 2019), where prominent researchers and experts from different disciplines such as education, psychology, data science, AI, and cognitive neuroscience, etc., exchanged their knowledge and ideas. This article is organized as follows: we start with an overview of recent progress of big data and AI in education. Then we present the major challenges and emerging trends. Finally, based on our discussions of big data and AI in education, conclusion and future scope are suggested.

Rapid advancements in big data and artificial intelligence (AI) technologies have had a profound impact on all areas of human society including the economy, politics, science, and education. Thanks in large part to these developments, we are able to continue many of our social activities under the COVID-19 pandemic. Digital tools, platforms, applications, and the communications among people have generated vast amounts of data (‘big data’) across disparate locations. Big data technologies aim at harnessing the power of extensive data in real-time or otherwise (Daniel, 2019). The characteristic attributes of big data are often referred to as the four V’s. That is, volume (amount of data), variety (diversity of sources and types of data), velocity (speed of data transmission and generation), and veracity (the accuracy and trustworthiness of data) (Laney, 2001; Schroeck et al., 2012; Geczy, 2014). Recently, a 5th V was added, namely value (i.e., that data could be monetized; Dijcks, 2013). Because of intrinsic big data characteristics (the five Vs), large and complex datasets are impossible to process and utilize by using traditional data management techniques. Hence, novel and innovative computational technologies are required for the acquisition, storage, distribution, analysis, and management of big data (Lazer et al., 2014; Geczy, 2015). Big data analytics commonly encompasses the processes of gathering, analyzing, and evaluating large datasets. Extraction of actionable knowledge and viable patterns from data are often viewed as the core benefits of the big data revolution (Mayer-Schönberger and Cukier, 2013; Jagadish et al., 2014). Big data analytics employ a variety of technologies and tools, such as statistical analysis, data mining, data visualization, text analytics, social network analysis, signal processing, and machine learning (Chen and Zhang, 2014).

As a subset of AI, machine learning focuses on building computer systems that can learn from and adapt to data automatically without explicit programming (Jordan and Mitchell, 2015). Machine learning algorithms can provide new insights, predictions, and solutions to customize the needs and circumstances of each individual. With the availability of large quantity and high-quality input training data, machine learning processes can achieve accurate results and facilitate informed decision making (Manyika et al., 2011; Gobert et al., 2012, 2013; Gobert and Sao Pedro, 2017). These data-intensive, machine learning methods are positioned at the intersection of big data and AI, and are capable of improving the services and productivity of education, as well as many other fields including commerce, science, and government.

Regarding education, our main area of interest here, the application of AI technologies can be traced back to approximately 50 years ago. The first Intelligent Tutoring System “SCHOLAR” was designed to support geography learning, and was capable of generating interactive responses to student statements (Carbonell, 1970). While the amount of data was relatively small at that time, it was comparable to the amount of data collected in other traditional educational and psychological studies. Research on AI in education over the past few decades has been dedicated to advancing intelligent computing technologies such as intelligent tutoring systems (Graesser et al., 2005; Gobert et al., 2013; Nye, 2015), robotic systems (Toh et al., 2016; Anwar et al., 2019), and chatbots (Smutny and Schreiberova, 2020). With the breakthroughs in information technologies in the last decade, educational psychologists have had greater access to big data. Concretely speaking, social media (e.g., Facebook, Twitter), online learning environments [e.g., Massive Open Online Courses (MOOCs)], intelligent tutoring systems (e.g., AutoTutor), learning management systems (LMSs), sensors, and mobile devices are generating ever-growing amounts of dynamic and complex data containing students’ personal records, physiological data, learning logs and activities, as well as their learning performance and outcomes (Daniel, 2015). Learning analytics, described as “the measurement, collection, analysis, and reporting of data about learners and their contexts, for purposes of understanding and optimizing learning and the environments in which it occurs” (Long and Siemens, 2011, p. 34), are often implemented to analyze these huge amounts of data (Aldowah et al., 2019). Machine learning and AI techniques further expand the capabilities of learning analytics (Zawacki-Richter et al., 2019). The essential information extracted from big data could be utilized to optimize learning, teaching, and administration (Daniel, 2015). Hence, research on big data and AI is gaining increasing significance in education (Johnson et al., 2011; Becker et al., 2017; Hwang et al., 2018) and psychology (Harlow and Oswald, 2016; Yarkoni and Westfall, 2017; Adjerid and Kelley, 2018; Cheung and Jak, 2018). Recently, the adoption of big data and AI in the psychology of learning and teaching has been trending as a novel method in cutting-edge educational research (Daniel, 2015; Starcic, 2019).

## The Position Formulation

A growing body of literature has attempted to uncover the value of big data at different education levels, from preschool to higher education (Chen N.-S. et al., 2020). Several journal articles and book chapters have presented retrospective descriptions and the latest advances in the rapidly expanding research area from different angles, including systematic literature review (Zawacki-Richter et al., 2019; Quadir et al., 2020), bibliometric study (Hinojo-Lucena et al., 2019), qualitative analysis (Malik et al., 2019; Chen L. et al., 2020), and social network analysis (Goksel and Bozkurt, 2019). More details can be found in the previously mentioned reviews. In this paper, we aim at presenting the current progress of the application of big data and AI in education. By and large, the research on the learner side is devoted to identifying students’ learning and affective behavior patterns and profiles, improving methods of assessment and evaluation, predicting individual students’ learning performance or dropouts, and providing adaptive systems for personalized support (Papamitsiou and Economides, 2014; Zawacki-Richter et al., 2019). On the teacher side, numerous studies have attempted to enhance course planning and curriculum development, evaluation of teaching, and teaching support (Zawacki-Richter et al., 2019; Quadir et al., 2020). Additionally, teacher dashboards, such as Inq-Blotter, driven by big data techniques are being used to inform teachers’ instruction in real time while students simultaneously work in Inq-ITS (Gobert and Sao Pedro, 2017; Mislevy et al., 2020). Big data technologies employing learning analytics and machine learning have demonstrated high predictive accuracy of students’ academic performance (Huang et al., 2020). Only a small number of studies have focused on the effectiveness of learning analytics programs and AI applications. However, recent findings have revealed encouraging results in terms of improving students’ academic performance and retention, as well as supporting teachers in learning design and teaching strategy refinement (Viberg et al., 2018; Li et al., 2019; Sonderlund et al., 2019; Mislevy et al., 2020).

Despite the growing number of reports and methods outlining implementations of big data and AI technologies in educational environments, we see a notable gap between contemporary technological capabilities and their utilization for education. The fast-growing education industry has developed numerous data processing techniques and AI applications, which may not be guided by current theoretical frameworks and research findings from psychology of learning and teaching. The rapid pace of technological progress and relatively slow educational adoption have contributed to the widening gap between technology readiness and its application in education (Macfadyen, 2017). There is a pressing need to reduce this gap and stimulate technological adoption in education. This work presents varying viewpoints and their controversial issues, contemporary research, and prospective future developments in adoption of big data and AI in education. We advocate an interdisciplinary approach that encompasses educational, technological, and governmental spheres of influence. In the educational domain, there is a relative lack of knowledge and skills in AI and big data applications. On the technological side, few data scientists and AI developers are familiar with the advancements in education psychology, though this is changing with the advent of graduate programs at the intersection of Learning Sciences and Computer Science. Finally, in terms of government policies, the main challenges faced are the regulatory and ethical dilemmas between support of educational reforms and restrictions on adoptions of data-oriented technologies.

## An Interdisciplinary Approach to Educational Adoption of Big Data and AI

**FIGURE 1:**
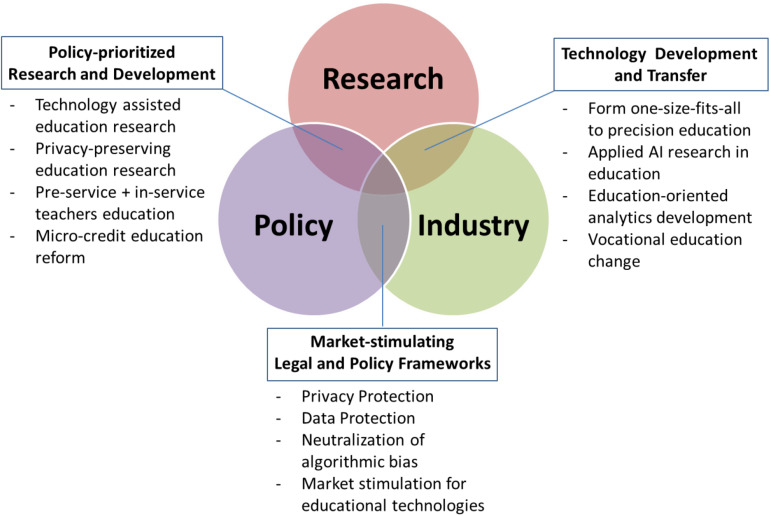
Contemporary developments and future trends at the intersections between research, policy, and industry driven by big data and AI advances in education.

In response to the new opportunities and challenges that the big data explosion and AI revolution are bringing, academics, educators, policy-makers, and professionals need to engage in productive collaboration. They must work together to cultivate our learners’ necessary competencies and essential skills important for the 21st century work, driven by the knowledge economy (Bereiter, 2002). Collaboration across diverse disciplines and sectors is a demanding task—particularly when individual sides lack a clear vision of their mutually beneficial interests and the necessary knowledge and skills to realize that vision. We highlight several overlapping spheres of interest at the intersection of research, policy-making, and industry engagements. Researchers and the industry would benefit from targeted educational technology development and its efficient transfer to commercial products. Businesses and governments would benefit from legislature that stimulates technology markets while suitably protecting data and users’ privacy. Academics and policy makers would benefit from prioritizing educational reforms enabling greater adoption of technology-enhanced curricula. The recent developments and evolving future trends at intersections between researchers, policy-makers, and industry stakeholders arising from advancements and deployments of big data and AI technologies in education are illustrated in Figure 1.

The constructive domains among stakeholders progressively evolve along with scientific and technological developments. Therefore, it is important to reflect on longer-term projections and challenges. The following sections highlight the novel challenges and future directions of big data and AI technologies at the intersection of education research, policy-making, and industry.

## Big Data and AI in Education: Research

An understanding of individual differences is critical for developing pedagogical tools to target specific students and to tailor education to individual needs at different stages. Intelligent educational systems employing big data and AI techniques are capable of collecting accurate and rich personal data. Data analytics can reveal students’ learning patterns and identify their specific needs (Gobert and Sao Pedro, 2017; Mislevy et al., 2020). Hence, big data and AI have the potential to realize individualized learning to achieve precision education (Lu et al., 2018). We see the following emerging trends, research gaps, and controversies in integrating big data and AI into education research so that there is a deep and rigorous understanding of individual differences that can be used to personalize learning in real time and at scale.

(1) Education is progressively moving from a one-size-fits-all approach to precision education or personalized learning (Lu et al., 2018; Tsai et al., 2020). The one-size-fits-all approach was designed for average students, whereas precision education takes into consideration the individual differences of learners in their learning environments, along with their learning strategies. The main idea of precision education is analogous to “precision medicine,” where researchers harvest big data to identify patterns relevant to specific patients such that prevention and treatment can be customized. Based on the analysis of student learning profiles and patterns, precision education predicts students’ performance and provides timely interventions to optimize learning. The goal of precision education is to improve the diagnosis, prediction, treatment, and prevention of learning outcomes (Lu et al., 2018). Contemporary research gaps related to adaptive tools and personalized educational experiences are impeding the transition to precision education. Adaptive educational tools and flexible learning systems are needed to accommodate individual learners’ interaction, pace, and learning progress, and to fit the specific needs of the individual learners, such as students with learning disabilities (Xie et al., 2019; Zawacki-Richter et al., 2019). Hence, as personalized learning is customized for different people, researchers are able to focus on individualized learning that is adaptive to individual needs in real time (Gobert and Sao Pedro, 2017; Lu et al., 2018).
(2) The research focus on deploying AI in education is gradually shifting from a computational focus that demonstrates use cases of new technology to cognitive focus that incorporates cognition in its design, such as perception (VanRullen, 2017), emotion (Song et al., 2016), and cognitive thinking (Bramley et al., 2017). Moreover, it is also shifting from a single domain (e.g., domain expertise, or expert systems) to a cross-disciplinary approach through collaboration (Spikol et al., 2018; Krouska et al., 2019) and domain transfers (L’heureux et al., 2017). These controversial shifts are facilitating transitions from the knowing of the unknown (gaining insights through reasoning) to the unknown of the unknown (figuring out hidden values and unknown results through algorithms) (Abed Ibrahim and Fekete, 2019; Cutumisu and Guo, 2019). In other words, deterministic learning, aimed at deductive/inductive reasoning and inference engines, predominated in traditional expert systems and old AI. Whereas, today, dynamic and stochastic learning, the outcome of which involves some randomness and uncertainty, is gradually becoming the trend in modern machine learning techniques.
(3) The format of machine-generated data and the purpose of machine learning algorithms should be carefully designed. There is a notable gap between theoretical design and its applicability. A theoretical model is needed to guide the development, interpretation, and validation of algorithms (Gobert et al., 2013; Hew et al., 2019). The outcomes of data analytics and algorithmically generated evidence must be shared with educators and applied with caution. For instance, efforts to algorithmically detect mental states such as boredom, frustration, and confusion (Baker et al., 2010) must be supported by the operational definitions and constructs that have been prudently evaluated. Additionally, the affective data collected by AI systems should take into account the cultural differences combined with contextual factors, teachers’ observations, and students’ opinions (Yadegaridehkordi et al., 2019). Data need to be informatively and qualitatively balanced, in order to avoid implicit biases that may propagate into algorithms trained on such data (Staats, 2016).
(4) There are ethical and algorithmic challenges when balancing human provided learning and machine assisted learning. The significant influence of AI and contemporary technologies is a double-edged sword (Khechine and Lakhal, 2018). On the one hand, it facilitates better usability and drives progress. On the other, it might lead to the algorithmic bias and loss of certain essential skills among students who are extensively relying on technology. For instance, in creativity- or experience-based learning, technology may even become an obstacle to learning, since it may hinder students from attaining first-hand experiences and participating in the learning activities (Cuthbertson et al., 2004). Appropriately balancing the technology adoption and human involvement in various educational contexts will be a challenge in the foreseeable future. Nonetheless, the convergence of human and machine learning has the potential for highly effective teaching and learning beyond the simple “sum of the parts of human and artificial intelligence” (Topol, 2019).
(5) Algorithmic bias is another controversial issue (Obermeyer et al., 2019). Since modern AI algorithms extensively rely on data, their performance is governed solely by data. Algorithms adapt to inherent qualitative and quantitative characteristics of data. For example, if data is unbalanced and contains disproportionately better information on students from general population in comparison to minorities, the algorithms may produce systematic and repeatable errors disadvantaging minorities. These controversial issues need to be addressed before its wide implementation in education practice since every single student is precious. More rigorous studies and validation in real learning environments are required though work along these lines is being done (Sao Pedro et al., 2013).
(6) The fast expansion of technology and inequalities of learning opportunities has aroused great controversies. Due to the exponential nature of technological progress, particularly big data and AI revolution, a fresh paradigm and new learning landscape are on the horizon. For instance, the elite smartphone 10 years ago, in 2010, was BlackBerry. Today, 10 years later, even in sub-Saharan Africa, 75% of the population has mobile phones several generations more advanced (GSMA Intelligence, 2020). Hence, the entry barriers are shifting from the technical requirements to the willingness of and/or need for adoption. This has been clearly demonstrated during the COVID-19 pandemic. The need for social distancing and continuing education has led to online/e-learning deployments within months (United Nations, 2020). A huge amount of learning data is created accordingly. The extraction of meaningful patterns and the discovery of knowledge from these data is expected to be carried out through learning analytics and AI techniques. Inevitably, the current learning cultures, learning experiences, and classroom dynamics are changing as “we live algorithmic lives” (Bucher, 2018). Thus, there is a critical need to adopt proper learning theories of educational psychology and to encourage our learners to be active participants rather than passive recipients or merely tracked objects (Loftus and Madden, 2020). For example, under the constructionist framework (Tsai, 2000), the technology-enhanced or AI-powered education may empower students to know their learning activities and patterns, predict their possible learning outcomes, and strategically regulate their learning behavior (Koh et al., 2014; Loftus and Madden, 2020). On the other hand, in the era of information explosion and AI revolution, the disadvantaged students and developing countries are indeed facing a wider digital divide. To reduce the inequalities and bring more opportunities, cultivating young people’s competencies is seemed like one of the most promising means (UNESCO, 2015). Meanwhile, overseas support from international organizations such as World Bank and UNESCO are imperative for developing countries in their communication infrastructure establishment (e.g., hardware, software, connectivity, electricity). Naturally, technology will not replace or hinder human learning; rather, a smart use of new technologies will facilitate transfer and acquisition of knowledge (Azevedo et al., 2019).

An overarching theme from the above trends of research is that we need theories of cognitive and educational psychology to guide our understanding of the individual learner (and individual differences), in order to develop best tools, algorithms, and practices for personalized learning. Take, for example, VR (virtual reality) or AR (augmented reality) as a fast-developing technology for education. The industry has developed many different types of VR/AR applications (e.g., *Google Expeditions* with over 100 virtual field trips), but these have typically been developed in the views of the industry (see further discussion below) and may not be informed by theories and data from educational psychology about how students actually learn. To make VR/AR effective learning tools, we must separate the technological features from the human experiences and abilities (e.g., cognitive, linguistic, spatial abilities of the learner; see Li et al., 2020). For example, VR provides a high-fidelity 3D real-life virtual environment, and the technological tools are built on the assumption that 3D realism enables the learner to gain ‘perceptual grounding’ during learning (e.g., having access to visual, auditory, tactile experiences as in real world). Following the ‘embodied cognition’ theory (Barsalou, 2008), we should expect VR learning to yield better learning outcomes compared with traditional classroom learning. However, empirical data suggest that there are significant individual differences in that some students benefit more than others from VR learning. It may be that the individuals with higher cognitive and perceptual abilities need no additional visuospatial information (provided in VR) to succeed in learning. In any case, we need to understand how embodied experiences (provided by the technology) interact with different learners’ inherent abilities (as well as their prior knowledge and background) for the best application of the relevant technology in education.

## Big Data and AI in Education: Policy-Making

Following the revolution triggered by breakthroughs in big data and AI technology, policy-makers have attempted to formulate strategies and policies regarding how to incorporate AI and emerging technologies into primary, secondary, and tertiary education (Pedró et al., 2019). Major challenges must be overcome in order to suitably integrate big data and AI into educational practice. The following three segments highlight pertinent policy-oriented challenges, gaps, and evolving trends.

(1) In digitally-driven knowledge economies, traditional formal education systems are undergoing drastic changes or even a paradigm shift (Peters, 2018). Lifelong learning is quickly being adopted and implemented through online or project-based learning schemes that incorporate multiple ways of teaching (Lenschow, 1998; Sharples, 2000; Field, 2001; Koper and Tattersall, 2004). This new concept of continual education will require micro-credits or micro-degrees to sustain learners’ efforts (Manuel Moreno-Marcos et al., 2019). The need to change the scope and role of education will become evident in the near future (Williams, 2019). For example, in the next few years, new instruction methods, engagement, and assessment will need to be developed in formal education to support lifelong education. The system should be based on micro-credits or micro-degrees.
(2) Solutions for integrating cutting-edge research findings, innovative theory-driven curricula, and emerging technologies into students’ learning are evidently beneficial, and perhaps even ready for adoption. However, there is an apparent divergence between a large number of pre-service and in-service teachers and their willingness to support and adopt these emerging technologies (Pedró et al., 2019). Pre-service teachers have greater exposure to modern technologies and, in general, are more willing to adopt them. In-service teachers have greater practical experience and tend to more rely on it. To bridge the gap, effective teacher education programs and continuing education programs have to be developed and offered to support the adoption of these new technologies so that they can be implemented with fidelity (O’Donnell, 2008). This issue could become even more pressing to tackle in light of the extended period of the COVID-19 pandemic.
(3) A suitable legislative framework is needed to protect personal data from unscrupulous collection, unauthorized disclosure, commercial exploitation, and other abuses (Boyd and Crawford, 2012; Pardo and Siemens, 2014). Education records and personal data are highly sensitive. There are significant risks associated with students’ educational profiles, records, and other personal data. Appropriate security measures must be adopted by educational institutions. Commercial educational system providers are actively exploiting both legislative gaps and concealed data acquisition channels. Increasing numbers of industry players are implementing data-oriented business models (Geczy, 2018). There is a vital role to play for legislative, regulatory, and enforcing bodies at both the national and local levels. It is pertinent that governments enact, implement, and enforce privacy and personal data protection legislation and measures. In doing so, there is a need to strike a proper balance between desirable use of personal data for educational purposes and undesirable commercial monetization and abuse of personal data.

## Big Data and AI in Education: Industry

As scientific and academic aspects of big data and AI in education have their unique challenges, so does the commercialization of educational tools and systems (Renz et al., 2020). Numerous countries have attempted to stimulate innovation-based growth through enhancing technology transfer and fostering academia-industry collaboration (Huggins and Thompson, 2015). In the United States, this was initiated by the Bayh-Dole Act (Mowery et al., 2001). Building a reciprocal and sustained partnership is strongly encouraged. It facilitates technology transfers and strengthens the links between academia and the education industry. There are several points to be considered when approaching academia-industry collaboration. It is important that collaboration is mutually beneficial. The following points highlight the overlapping spheres of benefits for both educational commerce and academia. They also expose existing gaps and future prospects.

(1) Commercializing intelligent educational tools and systems that include the latest scientific and technological advances can provide educators with tools for developing more effective curricula, pedagogical frameworks, assessments, and programs. Timely release of educational research advances onto commercial platforms is desirable by vendors from development, marketing, and revenue perspectives (Renz and Hilbig, 2020). Implementation of the latest research enables progressive development of commercial products and distinctive differentiation for marketing purposes. This could also potentially solve the significant gap between what the industry knows and develops and what the academic research says with regard to student learning. Novel features may also be suitably monetized—hence, expanding revenue streams. The gaps between availability of the latest research and its practical adoption are slowing progress and negatively impacting commercial vendors. A viable solution is a closer alignment and/or direct collaboration between academia and industry.
(2) A greater spectrum of commercially and freely available tools helps maintain healthy market competition. It also helps to avoid monopolies and oligopolies that stifle innovation, limit choices, and damage markets for educational tools. Some well-stablished or free-of-charge platforms (e.g., Moodle, LMS) might show such potential of oligopolies during the COVID-19 pandemic. With more tools available on the market, educators and academics may explore novel avenues for improving education and research. New and more effective forms of education may be devised. For instance, multimodal virtual educational environments have high potential future prospects. These are environments that would otherwise be impossible in conventional physical settings (see previous discussion of VR/AR). Expanding educational markets and commerce should inevitably lead to expanding resources for research and development funding (Popenici and Kerr, 2017). Collaborative research projects sponsored by the industry should provide support and opportunities for academics to advance educational research. Controversially, in numerous geographies there is a decreasing trend in collaborative research. To reverse the trend, it is desirable that academic researchers and industry practitioners increase their engagements via mutual presentations, educations, and even government initiatives. All three stakeholders (i.e., academia, industry, and government) should play more active roles.
(3) Vocational and practical education provides numerous opportunities for fruitful academia-industry collaboration. With the changing nature of work and growing technology adoption, there is an increasing demand for radical changes in vocational education—for both teachers and students (World Development and Report, 2019). Domain knowledge provided by teachers is beneficially supplemented by AI-assisted learning environments in academia. Practical skills are enhanced in industrial environments with hands-on experience and feedback from both trainers and technology tools. Hence, students benefit from acquiring domain knowledge and enhancing their skills via interactions with human teachers and trainers. Equally, they benefit from gaining the practical skills via interactions with simulated and real-world technological environments. Effective vocational training demands teachers and trainers on the human-learning side, and AI environments and actual technology tools on machine-learning side. Collaboration between academia and industry, as well as balanced human and machine learning approaches are pertinent for vocational education.

## Discussion and Conclusion

Big data and AI have enormous potential to realize highly effective learning and teaching. They stimulate new research questions and designs, exploit innovative technologies and tools in data collection and analysis, and ultimately become a mainstream research paradigm (Daniel, 2019). Nonetheless, they are still fairly novel and unfamiliar to many researchers and educators. In this paper, we have described the general background, core concepts, and recent progress of this rapidly growing domain. Along with the arising opportunities, we have highlighted the crucial challenges and emerging trends of big data and AI in education, which are reflected in educational research, policy-making, and industry. Table 1 concisely summarizes the major challenges and possible solutions of big data and AI in education. In summary, future studies should be aimed at theory-based precision education, incorporating cross-disciplinary application, and appropriately using educational technologies. The government should be devoted to supporting lifelong learning, offering teacher education programs, and protecting personal data. With regard to the education industry, reciprocal and mutually beneficial relationships should be developed in order to enhance academia-industry collaboration.

**TABLE 1:** Major challenges and possible solutions for integrating big data and AI into education.

**Aspect**	**Major challenges**	**Possible solutions**
Research	• The mode of education is progressively moving from a one-size-fits-all approach to precision education and individualized learning. • AI research in education is currently focused on intelligent computing technologies in a single domain. • The format, purpose, and meaning of machine-generated data should be carefully designed. • The significant influence of AI and big data technologies is a double-edged sword.	• Adaptive educational tools and flexible learning systems will be needed to accommodate individual learners’ needs. • The research focus on deploying AI in education needs to incorporate theories of cognition and knowledge about individual differences in student learning. • A theoretical model is needed to guide the development, interpretation, and validation of algorithms. The data analytics must be applied with caution. • Future studies should be aimed at using educational technologies in the appropriate context tailored to the characteristics of individual learners.
Policy-making	• In digitally-driven knowledge economies, traditional formal education systems are undergoing drastic changes or even a paradigm shift. • A large number of pre-service and in-service teachers are not ready to support and adopt new technologies. • There is a pressing need for privacy and personal data protections against unauthorized disclosure, commercial exploitation, and other abuses.	• New methods of instruction, engagement, and assessment will need to be developed in formal education to support lifelong education systems based on micro-credits or micro-degrees. • Effective teacher education and continuing education programs have to be designed and offered to support the adoption of these new technologies. • The government must seek an optimal balance between personal data collection and personal data protection in policy-making, implementation, and enforcement.
Industry	• The commercialization of intelligent educational tools and systems presents a set of difficult challenges. • Expanding spectrum of commercially and freely available tools is necessary to maintain healthy market competition. • Vocational and practical trainings need radical changes to remain relevant and prudent.	• Building a reciprocal and sustained partnership between academia and the education industry is strongly encouraged. • Collaborative research projects sponsored by the industry should provide support for academics to advance applied research and its commercialization. • Closer academia-industry collaboration with balanced human-oriented and machine-assisted learning.

Regarding the future development of big data and AI, we advocate an in-depth dialog between the supporters of “cold” technology and “warm” humanity so that users of technology can benefit from its capacity and not see it as a threat to their livelihood. An equally important issue is that overreliance on technology may lead to an underestimation of the role of humans in education. Remember the fundamental role of schooling: the school is a great equalizer as well as a central socialization agent. We need to better understand the role of social and affective processing (e.g., emotion, motivation) in addition to cognitive processing in student learning successes (or failures). After all, human learning is a social behavior, and a number of key regions in our brains are wired to be socially engaged (see Li and Jeong, 2020 for a discussion).

It has been estimated that approximately half of the current routine jobs might be automated in the near future (Frey and Osborne, 2017; World Development and Report, 2019). However, the teacher’s job could not be replaced. The teacher-student relationship is indispensable in students’ learning, and inspirational in students’ personal growth (Roorda et al., 2011; Cheng and Tsai, 2019). On the other hand, new developments in technologies will enable us to collect and analyze large-scale, multimodal, and continuous real-time data. Such data-intensive and technology-driven analysis of human behavior, in real-world and simulated environments, may assist teachers in identifying students’ learning trajectories and patterns, developing corresponding lesson plans, and adopting effective teaching strategies (Klašnja-Milicevic et al., 2017; Gierl and Lai, 2018). It may also support teachers in tackling students’ more complex problems and cultivating students’ higher-order thinking skills by freeing the teachers from their monotonous and routine tasks (Li, 2007; Belpaeme et al., 2018). Hence, it is now imperative for us to embrace AI and technology and prepare our teachers and students for the future of AI-enhanced and technology-supported education.

The adoption of big data and AI in learning and teaching is still in its infancy and limited by technological and mindset challenges for now; however, the convergence of developments in psychology, data science, and computer science shows great promise in revolutionizing educational research, practice, and industry. We hope that the latest achievements and future directions presented in this paper will advance our shared goal of helping learners and teachers pursue sustainable development.

## Author Contributions

HLu wrote the initial draft of the manuscript. PG, HLa, JG, and PL revised the drafts and provided theoretical background. SY, HO, JB, and RG contributed content for the original draft preparation of the manuscript. C-CT provided theoretical focus, design, draft feedback, and supervised throughout the research. All authors contributed to the article and approved the submitted version.

## Conflict of Interest

JG was employed by company Apprendis, LLC, Berlin. The remaining authors declare that the research was conducted in the absence of any commercial or financial relationships that could be construed as a potential conflict of interest.
